# Can dexamethasone improve postoperative sleep and postoperative delirium in elderly patients undergoing robot-assisted laparoscopic radical prostatectomy? Protocol for a prospective, randomized, double-blind, controlled study

**DOI:** 10.1186/s13063-023-07521-8

**Published:** 2023-08-08

**Authors:** Yaping Shi, Qingyu Sun, Yue Wang, Chunting Chen, Jianfei Jin, Wei Wang, Yuting Lu, Yi Hua, Jianming Liu, Jinjun Bian, Zhou Yi

**Affiliations:** 1 Department of Anesthesiology, the First Affiliated Hospital of Naval Medical University, Shanghai, People’s Republic of China; 2 Department of Anesthesiology, Shanghai Pulmonary Hospital, School of Medicine, Tongji University, Shanghai, China

**Keywords:** Dexamethasone, Robot-assisted radical prostatectomy, Elderly patient, Enhanced recovery after surgery, Postoperative delirium, Perioperative sleep disorders

## Abstract

**Background:**

Perioperative sleep disorders (PSD) are an independent risk factor for postoperative delirium (POD), which is a common complication after surgery. Elderly patients who undergo robot-assisted radical prostatectomy (RARP) often experience perioperative sleep disorders (PSD). Dexamethasone, a medication that works by inhibiting the hypothalamic-pituitary-suprarenal cortical axis, can reduce the negative effects of surgical stress. The objective of this study was to determine whether intravenous administration of dexamethasone at the time of anesthesia induction could improve postoperative sleep quality in elderly patients, thereby indirectly reducing the risk of postoperative cognitive impairment and accelerating postoperative rehabilitation.

**Methods/design:**

This study is a randomized, double-blind, placebo-controlled trial that was conducted at a single center. A sample size of 116 patients was determined through calculation, and these patients were randomly assigned to either the dexamethasone group (group D, *n* = 58) or the blank control group (group C, *n* = 58). On the day of surgery, the anesthesia nurse prepared either diluted dexamethasone or saline in advance, according to the patient's assigned group. The blinded anesthesiologist administered the medication during induction, and a dedicated person followed up with the patient for three consecutive postoperative days. All other aspects of care were managed equally between the two groups. The primary outcome measure was sleep quality, while secondary outcome measures included postoperative sleep time, postoperative delirium (POD), pain scores, and other complications. Relevant test measures were recorded for analysis.

**Discussion:**

This study aims to investigate the impact of intravenous dexamethasone on sleep quality and duration of patients undergoing robot-assisted radical prostatectomy (RARP). If the findings of this study protocol are affirmative, it could enhance the sleep quality of elderly patients after surgery, thereby minimizing the risk of postoperative delirium (POD), and providing substantial evidence for the perioperative enhanced recovery management of elderly patients.

**Trial registration:**

Chinese clinical trial registry: ChiCTR2200063488, Registered on 5 October 2022.

**Supplementary Information:**

The online version contains supplementary material available at 10.1186/s13063-023-07521-8.

## Background

Prostate cancer is the second most commonly diagnosed cancer and the sixth leading cause of cancer-related death in males. It is the most prevalent male urinary tumor in economically developed countries, and its incidence is higher in elderly patients who often have coexisting medical conditions [1]. Enhanced recovery after surgery (ERAS) interventions are widely adopted in general surgery, hepatobiliary surgery, and gynecology. However, there is limited research on the effectiveness of ERAS interventions in elderly patients [2, 3]. Postoperative sleep disturbance (PSD) is a common complication following surgery. In China, sleep disorders affect around 46.0% of elderly individuals, with 40.0% prevalence in elderly males. This is a frequent symptom among the elderly population [4]. Chronic sleep deprivation (SD) can impair the recovery and treatment of elderly patients with underlying medical conditions, as well as increase the risk of cardiovascular and cerebrovascular diseases, such as hypertension and cerebral infarction. It can also lead to postoperative delirium (POD), which is a significant risk factor for poor postoperative rehabilitation in the elderly [5]. Patients frequently experience severe sleep disorders during the perioperative period, which can be attributed to factors such as surgical environment, pain, and anxiety [6]. Therefore, it is crucial to focus on preventing and treating sleep disorders in the elderly during the perioperative period. Research has indicated that age is a significant risk factor for postoperative delirium (POD) [7, 8]. Moreover, various degrees of sleep deprivation, disruption, and abnormal sleep patterns before surgery can increase the incidence of POD [9].

Abnormal sleep patterns have been identified as an independent risk factor for postoperative delirium [10]. Improving sleep can help reduce the incidence of postoperative delirium.

Research has demonstrated that dexamethasone can diminish cortisol secretion via negative feedback inhibition of the hypothalamus-pituitary-suprarenal gland cortical axis, which could potentially ameliorate a range of negative effects caused by surgical stress and improve sleep quality [6, 11]. Other studies have indicated that preoperative administration of dexamethasone may lower the incidence of POD [12]. Additionally, some studies have reported that elevated serum cortisol levels in the early postoperative period are linked to a heightened risk of cognitive impairment [7, 13]. Furthermore, by diminishing cortisol secretion via negative feedback inhibition of the hypothalamus-pituitary-suprarenal gland cortical axis, dexamethasone can mitigate a variety of negative effects caused by surgical stress, subsequently improving postoperative insomnia and other symptoms [14, 15]. Our preliminary experiment revealed that dexamethasone has the potential to decrease inflammation, alleviate pain, and improve postoperative sleep. Furthermore, our findings indicate that there are no established guidelines for the clinical use of dexamethasone, and most anesthesiologists administer it based on their personal preferences. Therefore, we aim to investigate whether administering dexamethasone during anesthesia induction can ameliorate postoperative delirium, improve sleep quality among elderly patients after RARP, and ultimately facilitate enhanced recovery after surgery (ERAS).

## Methods

### Trial design

This trial is a single-center, randomized, double-blind, and placebo-controlled study. The study is structured as a parallel grouping with an allocation ratio of 1:1, embracing the framework of a superiority trial.

Changhai Hospital, located in Shanghai, China, is a comprehensive medical institution that integrates clinical practice, teaching, and research. Its Urology Department is among the top-ranking centers in China and is equipped with the most state-of-the-art Da Vinci surgical robot worldwide. Each year, a large number of patients flock to the hospital, drawn by its reputation. Patients who meet the specified criteria will be recruited through recruitment advertisements, with the deadline for recruitment projected to be June 31, 2024. Competent anesthesiologists in specialized preoperative evaluation clinics and wards will be responsible for selecting patients scheduled to undergo robot-assisted laparoscopic radical prostatectomy. We anticipate achieving a recruitment rate of 150%, with an estimated enrollment of 20 patients per month. The expected duration for recruitment is one year. The details of the research plan will not be disclosed to any previously involved medical personnel, and the exclusive responsibility for patient recruitment will be entrusted to specialized anesthesiologists. Patients who fail to meet the inclusion criteria will be excluded. Furthermore, patients who enroll in the trial have the right to discontinue participation and withdraw at any time. If a patient chooses to participate in the trial, they must provide a complete medical history and answer the investigator's questions truthfully. The patient's personal data will be safely stored and protected by the investigator. The trial is expected to be completed by December 2024. Patients will receive detailed information about the study's purpose, risks, and benefits prior to surgery, and must provide written informed consent before initiation of the study. The investigators will complete a case report form (CRF) that includes age (in years), height, weight, and the American Society of Anesthesiologists (ASA) classification. This reported protocol was in accordance with the Standard Protocol Items: Recommendations for Intervention Trials (SPIRIT) guidelines [16].

### Registration and ethics approval

On July 27th, 2022, the ethics committee of Shanghai Changhai Hospital granted approval for the trial under the number CHEC2022-132. Furthermore, the trial was registered with the Chinese Clinical Trial Registry under the registration number ChiCTR2200063488.

### Inclusion criteria

A) Participants underwent elective robotic-assisted laparoscopic radical prostatectomy;
B) Participants were 65 years of age or older;
C) Participants with American Society of Anesthesiologists (ASA) physical status classifications I to III were included;
D) Patients who had a Pittsburgh Sleep Quality Index (PSQI) score of ≥ 7 for nearly one month preoperatively were evaluated; A PSQI score of ≤ 6 indicated good sleep, while a score of ≥ 7 indicated the presence of a sleep disorder and eligibility for trial enrollment. (The PSQI total score ranges from 0 to 21, with higher scores indicating worse sleep quality.)
E) Participants who woke up before 5 PM were included; and
F) Participants had clear consciousness and normal communication ability.

### Exclusion criteria

A) Adrenal disease;
B) Severe hypertension, neuropsychiatric disease, and significant dysfunction of the cardiac, pulmonary, hepatic, and renal systems;
C) History of alcohol or substance abuse;
D) History of cerebral infarction or ICH within the last 6 months;
E) Long-term use of sedative or antidepressant medications; and
F) Preoperative exclusion criteria based on Mini-Mental State Examination (MMSE) score.

### Rejection criteria

Patients who meet any of the following criteria will be excluded from the study:

A) Patients who withdrew from the trial midway will be excluded from the study;
B) Patients who could not complete self-examination of sleep quality and cognitive assessment due to uncoordinated follow-up will be excluded from the study;
C) Patients who underwent secondary surgery will be excluded from the study.

### Sample size calculating

The sample size for this study was calculated based on the results of a previous study conducted by the research team, which has not yet been published. A total of 66 patients participated in the previous study. It was conducted at the same hospital by the same research team and the sample size was determined using the sample size calculation formula as described in the previous study. The control group had a score of 5.6 ± 2.0 while the 10 mg dexamethasone group had a score of 6.8 ± 1.6. The significance level (*α*) was set at 0.05 and the power (*β*) at 0.10. Accounting for a dropout rate of 20%, the final sample size was calculated to be 48 cases in each group, which would require approximately 58 cases per group and 116 cases in total. The formula for calculation is as follows.

$$n= \frac{{({Z}_{\alpha }+ {Z}_{\beta })}^{2} \left(1+1/k\right) {\sigma }^{2}}{{\delta }^{2}}$$

Analysis:

A) When *k* equals 1, both groups have the same sample size, and *n* represents the size of each group.
B) In our study, if α is 0.05 and *Z* is two-tailed, then *Z*_0.05_ is 1.96; if *β* is one-tailed and the test efficiency is 0.9, then *Z*_*β*_ equals 1.28.
C) The overall variance *σ*^2^ can be estimated using the sample variance *S*^2^ in the formula.
D) *δ* represents the difference between the average values of the two groups.

### Randomization and blinding

In order to mitigate selection bias, randomization and allocation concealment were conducted by a medical statistician prior to the commencement of the trial. The patients were randomly assigned to two groups in a 1:1 ratio using SPSS 26.0: the dexamethasone group (group D) and the control group (group C). Post-randomization information was sealed in 116 brown paper envelopes and stored in sequence in cabinets. On the day of surgery, the envelopes were opened in sequence based on group assignment and read by an anesthesia nurse on the research team. Dexamethasone or saline was prepared based on the randomization. An anesthesiologist who was blinded to the group assignments conducted the trial. A specialized anesthesiologist performed the outcome assessment, while another anesthesiologist carried out the data analysis. Both of them were unaware of the group allocations. Details regarding patient recruitment advertisement can be obtained from the corresponding author. Dr. Yaping Shi, a valued member of the research team, assumes the pivotal role of patient recruitment. Dr. Shi diligently arranges a preoperative visit with the patient the day prior to their scheduled surgery. During this essential preoperative encounter, her primary objective is to ensure that the patients possess a thorough understanding of the study and the clinical significance of their participation, while also obtaining their signed informed consent for the trial.

We will request consent for review of participants' medical records, and for the collection of blood samples to assess Hemoglobin, albumin, serum sodium, testosterone, cortisol, IL-6, TNF, NSE, and Sβ-100 were recorded before surgery, 1 day and 2 day after surgery. The patient's blood specimens will be collected by dedicated laboratory personnel. After proper processing, these samples will be stored in an ultra-low temperature freezer at − 80℃ for subsequent molecular biology experiments and surveillance.

### Experimental process

**Fig. 1:**
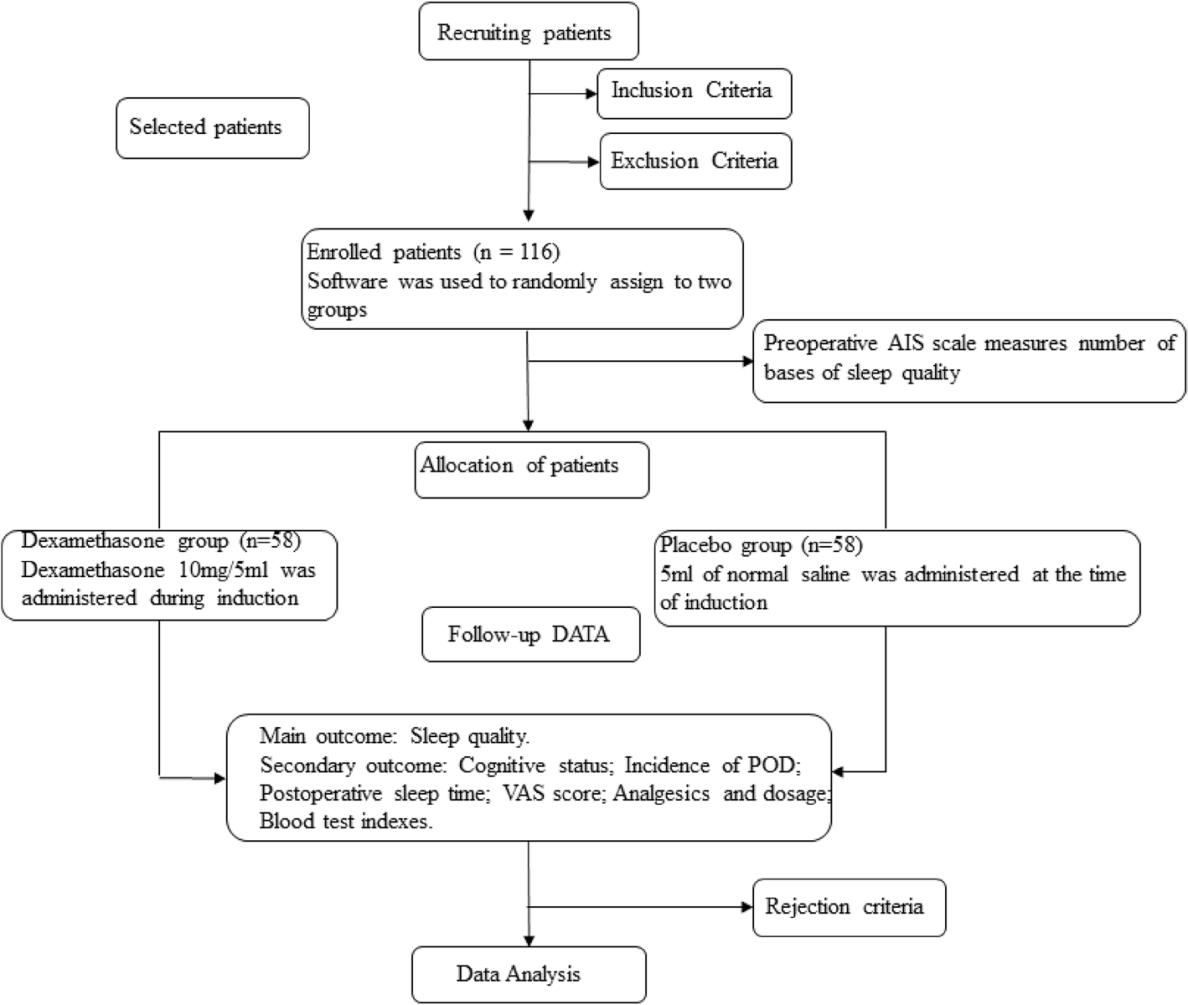
Presents a flowchart depicting the impact of dexamethasone on the quality of postoperative sleep and the incidence of postoperative delirium in elderly patients who have undergone radical prostatectomy

Before surgery, all recruited patients scheduled for elective robot-assisted radical prostatectomy were selected based on the inclusion criteria mentioned above, assessed using the PSQI scale, and screened for eligibility based on the exclusion criteria mentioned above. Standardized perioperative management and postoperative care process were uniformly implemented for the enrolled patients, which included preoperative preparation according to the standard surgical routine. The general flow chart and trial schedule are displayed in Fig. 1 and Table 1, respectively.

**Table 1:** Schedule

Events	Time						
	Preoperative			Intraoperative		Postoperative	
	*T*_0_		*T*_2_	*T*_3_	*T*_4_	*T*_6_	*T*_7_
**Enrollment**							
Eligibility screening		**X**					
Informed consent		**X**					
Random allocation		**X**					
**Interventions**							
Dexamethasone			**X**				
Normal saline			**X**				
**Data collation**							
Baseline variables		**X**	**X**	**X**	**X**	**X**	
Follow-up results						**X**	**X**
Case report form		**X**	**X**	**X**	**X**	**X**	**X**
Relevant test data		**X**					**X**
**Assessments**							
PSQI scale		**X**					
MMSE scale		**X**					
AIS scale		**X**					**X**
CAM scale							**X**
Blood test indexes		**X**				**X**	
Postoperative complications						**X**	**X**

T_0_: before admission to the operating room; T_2_: induction of anesthesia; T_3_: beginning of surgery; T_4_: end of surgery; T_6_: in the recovery room after the operation; T_7_: within 72 h after operation

Patients will be required to abstain from food and alcoholic beverages on the day of surgery. Upon admission, a venous access will be established for the patient and vital signs are monitored, including electrocardiogram (ECG), heart rate (HR), pulse oxygen saturation (SpO_2_), and invasive blood pressure (IBP), using radial artery catheterization. The test group received dexamethasone 10 mg/5 ml during induction, while the control group received normal saline 5 ml. Patients will be received pre-administered oxygen at 6 L/min, with an inspired oxygen concentration of 100%. Induction was achieved rapidly using intravenous bolus doses of propofol 1 ~ 3 mg/kg, sufentanil 0.3 ~ 0.5 μg/kg, and cis-atracurium besylate 0.2 mg/kg was rapidly induced, after the patient had lost consciousness and the muscle was fully relaxed. The trachea will be then intubated under visualization by the same attending anesthesiologist and confirmed to be in position. The anesthetic machine will be used for mechanical ventilation, and respiratory parameters were adjusted to maintain an oxygen concentration of 100% and an end-respiratory carbon dioxide level of 35 ~ 45 mmHg throughout the surgery. Appropriate muscle depth will be maintained throughout the surgery by continuous infusion of propofol 3 ~ 5 mg/(kg h)^−1^ and remifentanil (0.1 ~ 0.3 µg/(kg min)^−1^). Intravenous cisatracuride will be administered 30 min before the end of surgery, and a static push of sufentanil 0.1 ~ 0.2 µg/kg was given if appropriate. Vasoactive drugs will be administered as needed to maintain circulation. Regular neostigmine (2 mg) and atropine (0.5 mg) will be administered to antagonize neuromuscular blockade at the end of surgery. Postoperatively, the patient will be transferred to the post-anesthesia awakening recovery unit (PACU). For postoperative patient-controlled intravenous analgesia (PCIA), a sufentanil analgesic drug will be used at a rate of 1 µg/kg via both pumps at 2 ml/h, with a single additional dose of 0.5 ml and a lockout time of 15 min.

Follow-up visits will be conducted with registrars who were blinded to group assignment and medication. Data entry for nodal time observation indicators will be completed solely based on the CRF form. The patients’ general condition, such as gender, age, BMI, medical history, height, weight, and AIS score on the night before surgery, will be documented. The heart rate and mean arterial pressure will be measured at four time points: after admission to the operating room (*T*_1_), at the start (*T*_3_), and end (*T*_4_) of surgery, and 30 min after awakening (*T*_5_). The following parameters will be recorded: operative time, anesthesia time, intraoperative blood loss, intraoperative fluid volume, intraoperative dosage of sufentanil and remifentanil, type and dosage of intraoperative vasoactive drug, wake time (time from end of surgery to extubation), and VAS score for resting and coughing 30 min after awakening. All patients will be followed up by a designated person within 72 h after operation. Postoperative sleep quality, sleep duration, and continuity were assessed using the AIS scale and visual analog scale (VAS) score, and analgesic drug doses will be recorded at 24, 48, and 72 h after the operation. Cognitive function will be evaluated using the CAM assessment. Hemoglobin, albumin, serum sodium, testosterone, cortisol, IL-6, TNF, NSE, and Sβ-100 will be measured before surgery, and at 1 and 2 days post-operation.

### Outcomes

#### Primary outcome

The primary outcome measured was the quality of sleep 24 and 48 h following surgery. The Athens Insomnia Scale (AIS) was utilized to assess sleep quality. Scores on the AIS range from 0 to 24, with scores less than 4 indicating no sleep disorder, scores of 4 to 6 indicating a sleep CAM disorder, and scores greater than 6 indicating insomnia.

#### Secondary outcome

Secondary outcomes included postoperative sleep time, incidence of postoperative delirium (POD), VAS pain score, analgesic use and dosage, blood test indexes (hemoglobin, albumin, blood sodium, testosterone, cortisol, IL-6, TNF-α, NSE, and S β-100), and perioperative-related indicators (operation time, anesthesia time, intraoperative blood loss and fluid volume, sufentanil and remifentanil dosage, vasoactive drug type and dosage, and awakening time from the end of the operation to extubation time). The postoperative sleep time was assessed based on the patient's self-reported time of falling asleep to waking up. The CAM method was used for POD diagnosis, requiring positive scores for feature 1 plus 2 and feature 3 or 4 to be considered positive for CAM (Table 2). The use of analgesics and their dosage were recorded.

**Table 2:** Confusion assessment method (CAM)

Characteristic	Manifestations
Acute onset and fluctuating course	Whether there is evidence of an acute change in mental status compared with the patient’s basal level
	Whether the patient's (abnormal) behavior fluctuates over the course of 1 day (symptoms are sometimes absent or mild and sometimes severe)
Inattention	Whether patients have difficulty concentrating, such as attention is easily distracted or cannot keep up with the topic being talked about
Disorganized thinking	Whether the patient’s thinking is disorganized or incoherent, such as when the topic of conversation is scattered or irrelevant, thinking is unclear or illogical, or suddenly changes from one topic to another without warning
Altered level of consciousness	Whether the patient’s current level of consciousness is abnormal, such as hypervigilance (excessive sensitivity to environmental stimuli, startle easily), somnolence (drowsiness, easy to wake up), or coma (difficult to wake up)

Scoring criteria: delirium was diagnosed with characteristic 1 plus 2 and characteristic 3 or 4 positive = CAM positive

### Data management and trial oversight

The personal information and records of each participant shall be meticulously preserved in dedicated files and securely stored within a lockable cabinet. Electronic files, safeguarded by password protection, shall rest exclusively on Changhai Hospital’s computer systems. Access to all research data shall be granted solely to authorized members of the research team.

Upon completion of the trial, all participants will receive a comprehensive summary of the research findings as well as information regarding their respective groups. All personal information shall be expunged prior to the public disclosure of the trial results.

Data analysis will be conveyed to an independent Data Monitoring Committee (DMC) with utmost confidentiality, wherein this committee will be tasked with evaluating the cumulative trial data and other pertinent research findings to ascertain the justifiability of recruiting additional participants. It is within their purview to offer suggestions in this regard.

The DMC is scheduled to convene prior to the commencement of the trial and one month after the initiation of participant recruitment, thereafter convening on a monthly basis until the completion of participant enrollment.

Regulatory officials convene monthly meetings to review the conduct of the trial. In accordance with the specific risk assessment of the trial and the documentation in the monitoring plan, on-site monitoring will be conducted. Supervisors will be granted authorization to conduct trial-related monitoring, regulatory inspections, audits, and ethical reviews, while also being provided with direct access to source data/documents.

### Confidentiality

The gathered information will be shared within the project team's secure network drive, overseen by dedicated personnel responsible for data maintenance and privacy protection. Access will be restricted only to members of the experimental team. Participants will be assigned individual trial identification numbers. The experimental data will be published in the form of an article.

### Statistical analysis

We analyzed the data in this study using the statistical software SPSS 26.0. We presented statistical descriptions of the measurement data using either $\overline{\mathrm{x} }$±s or median and interquartile range. We performed comparisons between groups using either a two-sample t-test or Mann–Whitney *U* test. We statistically described counting data using rate and constituent ratio. We performed comparisons between groups using either the *χ*^2^ test or Fisher’s exact probability method for pairwise comparisons. We performed comparisons of rank data using the Mann–Whitney *U* test. We analyzed factors associated with POD using logistic regression analysis. In logistic regression analysis, we set α Into as 0.05 and α Out as 0.10. For other hypothesis tests, we considered *P* < 0.05 as statistically significant.

## Discussion

This study protocol follows a single-center, randomized, double-blind, placebo-controlled design. Our objective is to investigate the potential of administering dexamethasone during anesthesia induction to improve postoperative delirium in elderly patients undergoing robot-assisted radical prostatectomy (RARP), enhance sleep quality, and ultimately facilitate enhanced recovery after surgery (ERAS).

It has been shown that prostate cancer is a prevalent urinary tract neoplasm among elderly men, and early surgical intervention is the primary treatment modality [17]. In recent years, minimally invasive surgery techniques, such as robotic-assisted radical prostatectomy (RARP) and laparoscopic radical prostatectomy (LRP), have become increasingly popular, replacing traditional open surgery. However, despite its advantages over traditional surgery, RARP has not significantly reduced many of the anesthesia-related complications associated with surgery. Common postoperative problems among elderly patients undergoing RARP include pain, nausea, vomiting, sleep disturbance, and cognitive decline. Of these, sleep disturbance is particularly prevalent among the elderly and has not been effectively addressed to date.

Research has demonstrated that dexamethasone is an artificially synthesized corticosteroid with potent anti-inflammatory effects, that reduces cortisol secretion before stress stimulation via negative feedback inhibition of the hypothalamus–pituitary–adrenal cortex axis. This reduces the negative effects caused by surgical stress to some extent [18]. Other studies have indicated that preoperative administration of dexamethasone may lower the incidence of POD [12]. Additionally, some studies have reported that elevated serum cortisol levels in the early postoperative period are linked to a heightened risk of cognitive impairment [7, 13]. Therefore, the intravenous administration of dexamethasone during anesthesia induction theoretically has the potential to reduce the release of inflammatory mediators, attenuate the inflammatory response, decrease perioperative cortisol secretion, improve patient sleep quality, and reduce the incidence of postoperative delirium (POD).

There are several limitations in our experimental design. Firstly, patients undergoing robot-assisted radical prostatectomy will be recruited from various surgical teams at Changhai Hospital Urology Center. Due to differences in anatomical sites, surgical techniques, and surgical duration, the surgical trauma experienced by patients may vary, and all of these factors could potentially impact postoperative sleep quality. Secondly, whether patients received preoperative chemotherapy or radiation therapy may also be an important factor influencing postoperative sleep quality.

In our previous study on postoperative nausea and vomiting, we observed that the administration of dexamethasone improved the duration and quality of sleep in patients to some extent. Therefore, we established an experimental group for intravenous injection of dexamethasone in this trial to investigate whether the administration of dexamethasone during induction affects postoperative sleep quality and cognitive function. This study can also serve as a reference for improving the anesthesia program for elderly patients and for further research.

### Trial status

This study employs Protocol Version 2.0. After receiving ethics approval from the RMIT Human Research Ethics Committee (HREC), recruitment advertising for the study began in July 2022. Recruitment for this study is ongoing, and it is expected to conclude by June 2024.

### Supplementary Information

**Additional file 1.**

## Data Availability

The datasets analyzed during the current study and statistical code are available from the corresponding author on reasonable request, as is the full protocol.

## Abbreviations

PSD: Perioperative sleep disorders
POD: Postoperative delirium
RARP: Robot-assisted radical prostatectomy
ERAS: Enhanced recovery after surgery
ASA: American Society of Anesthesiologists
CRF: Case report form
PSQI: Pittsburgh Sleep Quality Index
MMSE: Mini-Mental State Examination
ECG: Electrocardiogram
HR: Heart rate
SpO_2_: Pulse oxygen saturation
IBP: Invasive blood pressure
PACU: Post anesthesia awakening recovery unit
PCIA: Patient controlled intravenous analgesia
AIS: Athens Insomnia Scale
CAM: Confusion assessment method
DMC: Data Monitoring Committee
VAS: Visual analog scale
