# Correction: Can dexamethasone improve postoperative sleep and postoperative delirium in elderly patients undergoing robot-assisted laparoscopic radical prostatectomy? Protocol for a prospective, randomized, double-blind, controlled study

**DOI:** 10.1186/s13063-023-07583-8

**Published:** 2023-10-01

**Authors:** Yaping Shi, Qingyu Sun, Yue Wang, Chunting Chen, Jianfei Jin, Wei Wang, Yuting Lu, Yi Hua, Jianming Liu, Jinjun Bian, Yi Zhou

**Affiliations:** 1 Department of Anesthesiology, the First Affiliated Hospital of Naval Medical University, Shanghai, People’s Republic of China; 2 Department of Anesthesiology, Shanghai Pulmonary Hospital, School of Medicine, Tongji University, Shanghai, China

**Correction: BMC Trials 24, 505 (2023)**

**https://doi.org/10.1186/s13063-023-07521-8**

Following publication of the original article [1], we have been informed that the name of one of the corresponding authors was incorrectly tagged as Zhou Yi instead of Yi Zhou.

At the same time, the affiliation of “Jianming Liu” should be “Department of Anesthesiology, Shanghai Pulmonary Hospital, School of Medicine, Tongji University, Shanghai, China”. In the original text, it was “Department of Anesthesiology, School of Medicine, Shanghai Pulmonary Hospital, Tongji University, Shanghai, People’s Republic of China”.

The original article has been corrected.
